# The development and psychometric properties of a measure of clinicians’ attitudes to depression: the revised Depression Attitude Questionnaire (R-DAQ)

**DOI:** 10.1186/s12888-014-0381-x

**Published:** 2015-02-05

**Authors:** Mark Haddad, Marco Menchetti, Eamonn McKeown, André Tylee, Anthony Mann

**Affiliations:** Centre for Mental Health Research, School of Health Sciences, City University London, Northampton Square, London, EC1V 0HB UK; Department of Medical and Surgical Sciences, University of Bologna, Bologna, Italy; Centre for Health Services Research, City University London, London, UK; Section of Primary Care Mental Health, Health Services and Population Research Department, King’s College London, London, UK

## Abstract

**Background:**

Depression is a common mental disorder associated with substantial disability. It is inadequately recognised and managed, and clinicians’ attitudes to this condition and its treatment may play a part in this. Most research in this area has used the Depression Attitude Questionnaire (DAQ), but analyses have shown this measure to exhibit problems in psychometric properties and suitability for the health professionals and settings where depression recognition may occur.

**Methods:**

We revised the DAQ using a pooled review of findings from studies using this measure, together with a Delphi study which sought the opinions of a panel of relevant experts based in the UK, USA, Australia, and European countries (n = 24) using 3 rounds of questioning to consider attitude dimensions, content, and item wording. After item generation, revision and consensus (agreement >70%) using the Delphi panel, the revised DAQ (R-DAQ) was tested with 1193 health care providers to determine its psychometric properties. Finally the test-retest reliability of the R-DAQ was examined with 38 participants.

**Results:**

The 22-item R-DAQ scale showed good internal consistency: Cronbach’s alpha coefficient was 0.84; and satisfactory test-retest reliability: intraclass correlation coefficient was 0.62 (95% C.I. 0.37 to 0.78). Exploratory factor analysis favoured a three-factor structure (professional confidence, therapeutic optimism/pessimism, and a generalist perspective), which accounted for 45.3% of the variance.

**Conclusions:**

The R-DAQ provides a revised tool for examining clinicians’ views and understanding of depression. It addresses important weaknesses in the original measure whilst retaining items and dimensions that appeared valid. This revised scale is likely to be useful in examining attitudes across the health professional workforce and beyond the confines of the UK, and may be valuable for the purpose of evaluating training that aims to address clinicians’ attitudes to depression. It incorporates key dimensions of attitudes with a modest number of items making it applicable to use in busy clinical settings.

**Electronic supplementary material:**

The online version of this article (doi:10.1186/s12888-014-0381-x) contains supplementary material, which is available to authorized users.

## Background

Depression is one of the most common mental disorders, affecting an estimated 350 million people worldwide [1] and with a 12-month community prevalence of 4% to 7% [2]. It is the most prominent risk factor for suicide [3] and its high prevalence coupled with negative effects on function and the likelihood of recurrence for around half of those affected mean it is currently the third leading cause of disease burden in the world, and the leading cause in middle- and high-income countries [4].

The prevalence and disabling effects of depression are increased among people with long-term medical conditions such as coronary heart disease and type 2 diabetes [5] and it is frequently encountered within primary care, where health professionals have a key role in identification and initial management and often serve as gatekeepers for ongoing care. Its recognition and management is hampered by a number of factors, including frequent comorbid presentations, patient apprehension about disclosing mental issues, organisational capacity, and the knowledge and attitudes of clinicians [6,7].

Exploration of public attitudes has revealed widespread stigma about mental illnesses including depression, involving a desire to maintain social distance, blame attributed to the person for their problems, fears about dangerousness, and pessimistic views about the potential for recovery [8]. Negative public attitudes are linked to beliefs held by people with mental illness that they are socially unacceptable or considered this way by others (self-stigma and perceived stigma), which lead to reduced help-seeking and problem disclosure, under-treatment and marginalization. A recent systematic review of attitude time trends over the past decade or more indicates that although there has been a general increase in the view that mental health problems require professional help, many negative stereotypes still persist [8].

The attitudes of health professionals are likely to influence patients’ problem disclosure and to be an important factor affecting initial problem identification in the clinical encounter and subsequent treatment decisions [9-12], as well as their willingness to adopt new approaches to this part of their clinical role. Clinicians’ views about mental illness may incorporate some of the stereotypes and misunderstandings pervasive in society, as well as being related to personal experiences and the professional training that they have undertaken. Examining and measuring health professionals’ attitudes to depression is important as it enables exploration of changes over time and between different groups and settings, so allowing greater understanding and more systematic evaluation of processes such as specific educational activities or wider mass campaign initiatives that might improve condition recognition and management.

The instrument most widely used to examine clinicians’ attitudes to depression is the ‘depression attitude questionnaire’ (DAQ), a 20-item scale which was developed in the UK [13] for General Practitioners (GPs), but has subsequently also been used with other professions such as psychiatrists [14], district nurses [15], school nurses [16], and pharmacists [17], as well as general hospital staff [18], with studies conducted in several European nations [19] as well as Brazil [20], Australia [21], several African nations [22] and Japan [23].

Despite its extensive use, a systematic review and pooled analysis of studies employing the DAQ revealed investigators’ application of differing factor structures and differing sub-scales, with modest internal consistency values for the overall scale and various sub-scales [19]. In part because of these uncertainties about the psychometric adequacy of the instrument, these research reports have often reported individual item scores in preference to using scale and sub-scale scores. Alongside concerns about the psychometric adequacy of the DAQ, are concerns that its construction for a specific professional group within a particular setting (UK primary care) during the early 1990s may limit its comprehensibility and applicability for use with other professional groups in the array of settings (both geographic and organisational) where depression recognition and management may be an important part of the health professional’s role. Consequently, a study to revise the DAQ was instigated. Its aims were to build upon the pooled analysis of DAQ study findings [19] to develop a modified tool with improved construct validity, internal consistency, reliability and readability, suitable for measuring clinicians’ attitudes across the primary care workforce and beyond the confines of the UK.

## Methods

### Research design

This study was approved by the National Hospital for Neurology & Neurosurgery Research Ethics Committee 09/H0716/56, and funded by a grant from the Institute of Social Psychiatry, a charitable grant-awarding body.

The study was conducted in two phases. First was an instrument development and revision phase: using the findings of the pooled analysis of DAQ studies [19] to provide initial indication of candidate items, a three-round Delphi method consensus exercise with an international expert panel comprised of 24 clinicians and academics was conducted to select and generate relevant items, evaluate face and content validity, and determine the most appropriate phrasing.

Second was a testing phase, involving a cross-sectional study with 1193 health professionals using an electronic self-report version of the 30-item draft questionnaire derived from the Delphi exercise. We conducted exploratory factor analysis, item-total correlations, and measures of scale and sub-scale internal consistency of the survey findings, enabling scale reduction resulting to a 22-item R-DAQ, which was finally examined for test-retest reliability with an independent sample of 38 health professionals.

### Development phase

The Delphi method was developed at the Rand Corporation in the 1950s and is a widely used technique for establishing consensus from experts within particular topic areas. It was initially used to assist forecasting the occurrence of future events, but is more commonly used within health and education research for the purposes of establishing core professional competencies, defining outcome measurements, validating content of measurement tools and critically assessing quality criteria [24]. Consensus-building is sought by using a series of (typically three) questionnaires to collect data from a panel of selected subjects. The first round may involve either an open-ended questionnaire to elicit initial information about the content area or a structured questionnaire based upon a review of the literature or existing tool. Successive questionnaire rounds are built iteratively upon the results of preceding rounds, with participants evaluating their level of agreement with items by reviewing their responses in relation to the summarised scores from the whole panel. A development of this approach, in which ratings of item relevance by content experts are used to provide a measure of the content relevance of an instrument, is widely used by scale developers with the resulting index of proportional agreement termed the content validity index (CVI) [25]. In this study, participants were requested to rate each of the potential questionnaire statements with a 5-point scale according to its usefulness for identifying clinicians’ attitudes to depression.

### Participants

For this study, an expert panel of health professional clinicians and academics who had led or participated in depression attitudes research was identified from a search of the published literature. Additionally, a search of the grey literature (using ISI Proceedings and the Department of Health National Research Register) together with contacts with academic institutes were also used to find unpublished or ongoing investigations. Thirty-two potential participants were identified and their involvement requested and informed consent obtained; they comprised academic and clinical nurses, GPs, psychiatrists, psychologists, social scientists and pharmacists, and they were based in the UK and other European countries, the USA, Australia and Taiwan.

### Development phase procedure and analysis

The first round questionnaire provided the study rationale and brief details of the pooled review of DAQ findings, and sought panellists’ ratings (level of agreement) concerning: the importance of measuring clinicians’ attitudes to depression; whether they perceived the DAQ to be adequate or there to be potential for its revision; and whether the DAQ visual analogue scale format (as opposed to a Likert type response format) should be retained.

In this first round, participants were asked to consider what dimensions of a clinician’s attitudes to depression are the most important to measure by ranking potential factors (by level of importance) derived from a review of DAQ studies and related scales, and to suggest any further dimensions or relevant underlying factors. Finally, within this initial questionnaire, they were asked to rate a total of 26 candidate items, 11 derived from the original DAQ on the basis of item performance in the pooled analysis (individual sampling adequacy, item-total correlations, and weak or complex cross-loadings in relation to meaningful factors) [19], and a further 15 items derived from other depression and mental illness attitude measures including the Defeat Depression Campaign Mori Poll questionnaire [26], European Alliance Against Depression EAAD instruments [27], and the survey used by the Department of Health within the Research Surveys of Great Britain (RSGB) Omnibus [28] and derived from the Community Attitudes Toward the Mentally Ill (CAMI) measure [29].

The panellists were requested to rate each of the statements with a 5-point scale, and were invited to provide suggestions for potential new items or for wording modifications of existing items. In the first round, the panellists’ ratings were according to each item’s *usefulness* (relevance to the purpose of examining a clinician’s to depression), *clarity* (wording, structure), and *equivalence* across settings and occupational groups (rather than specificity to particular countries/professional groups). In the subsequent rounds, the panellists’ ratings of retained and newly proposed items were only on the basis of their usefulness.

In this study, the panellist’s ratings for each item were dichotomised, with neutral ratings discounted, and a threshold of 70% of ratings (as either *very useful* or *useful*) was set for item retention to the next round. In line with widely accepted recommendations for assessing content validity where there are more than six expert raters, a mean value of 0.78 for item-level CVI threshold (i.e. the number of raters judging the item as useful divided by the total number of raters applying judgement) was set to determine inclusion in the testing version of the R-DAQ. Additionally the scale-level CVI was determined by summing the individual item CVI scores for all items meeting item-level threshold, and dividing by the number of items. In line with expert recommendations, a level of 0.90 was set as the standard for this index of average congruity [30].

### Testing phase

#### Provisional R-DAQ instrument

The testing version of the R-DAQ contained 30 items. A 5-point Likert scale was used and response options were 1 = Strongly disagree, 2 = Disagree, 3 = Neither agree nor disagree, 4 = Agree and 5 = Strongly agree.

Fifteen items required reverse scoring, and scores could range from 30 to 150 with a lower score indicating a more pejorative, negative, pessimistic and unconfident view of depression and its management.

### Procedure

#### Sample selection and size

The purpose of this study was the development and testing of a revised attitude measure. There are several ‘rules of thumb’ concerning the appropriate sample size for factor analytic studies such as between 5 and 10 respondents per item; and indeed the majority of such studies use subject to item ratios of 10:1 or less [31]. However, best practice guidance indicates that exploratory factor analysis is generally a ‘large sample procedure’ – although smaller samples may be appropriate where data exhibit uniformly high (0.8 or higher) communalities and factor loadings, with an absence of cross-loading and few (three or less) factors with a minimum of three and preferably five or more strongly loading items, this is uncommon in social science research [31,32]. Where analyses indicate four variables per factor, with widely ranging communalities (between 0.2 and 0.8), a minimum sample size of 900 is recommended, with markedly reduced sample requirements where there are more variables per factor [32]. We judged an adequate sample size to be around 600 or more participants. Rates of response to surveys of health professionals vary widely: reviews indicate a range from 16% to 91% [33]. We conservatively estimated a response rate for this e-survey of 10%, indicating a total sample of 6000 to be necessary to provide sufficient responses.

The questionnaire was made accessible online for self-completion and potential respondents were invited to participate and follow a link to this electronic questionnaire by a brief letter included in e-bulletins sent to a randomly selected sample of members of the Mental Health Nurses’ Forum and the Practice Nurses’ Forum of the Royal College of Nursing (RCN) (7,550 of the total RCN membership of 410,000), and the Royal College of General Practitioners Forum for Mental Health in Primary Care (a collaboration hosted jointly between the Royal College of Psychiatrists and the Royal College of General Practitioners with around 100 members). Invitations to participate were additionally sent to clinical research colleagues in Finland and Italy. As participation involved responding to an anonymised electronic survey which included participant information, informed consent was inferred by completion.

### Statistical analysis

#### Descriptives

All statistical analysis was carried out using SPSS version 17 [34]. The mean scores, standard deviation and distribution of response data for each item were examined for normality, using measures of skewness and kurtosis and QQ plots.

#### Construct validity

In order to evaluate sampling adequacy to perform a satisfactory factor analysis, the Kaiser-Meyer-Olkin (KMO) [35] measure of sampling adequacy for each item was examined using the anti-image of the correlation matrix, to determine whether all the measures of sampling were above the acceptable level of 0.5 [36]. The overall KMO statistic and the Bartlett test of sphericity [37] were applied. The former examines whether the items have enough in common to justify conducting a factor analysis, with desirable values closer to 1 in a range from 0 to 1, and a minimum recommended value of 0.6; whilst the latter is a chi square test of the null hypothesis of no relationship between the items (a significant result indicates suitability for analysis).

The dimensionality of the scale was determined by performing exploratory factor analysis (EFA) using principal axis factoring and oblique rotation (Direct Oblimin). Recent reviews have critically evaluated the approaches commonly used for EFA, noting that although Principle Components Analysis (PCA) and Varimax rotation are very widely used by health and social science researchers, factor analysis is likely to be a superior method in many cases, providing more accurate findings especially where there are low component loadings (0.40), and few items per component [32], whereas PCA can produce inflated values of variance accounted for by the components. Similarly, a reliance in the literature upon orthogonal (typically Varimax) rotation has been critiqued because oblique methods allow the factors to correlate, and because typically the factors examined by health and social scientists (such as psychological and social phenomena) are likely to be correlated, this method should theoretically render more accurate and reproducible solutions [31]. If the factors are truly uncorrelated, orthogonal and oblique rotations result in near-identical findings.

The criteria for determining the scale structure and number of factors were multi-faceted, involving use of the Kaiser criterion (that is, where the factors with eigenvalues greater than 1 are considered for retention) and Catell’s scree plot wherein the eigenvalues of the correlation matrix are plotted from largest to smallest and the number of factors to be included in the model are inferred based on those points before a drop (or elbow) is evident [38]. Additionally, the amount of variance explained by differing models, and factor loading equal to or greater than 0.3 were applied [31,39]. Items were removed for failure to load on any factor at this minimum threshold or for cross-loading on multiple factors with values ≥ 0.3 [31]. Overall, a simple and parsimonious set of latent constructs was sought, with judgments based on the interpretability of the factor structure underlying the item variables, and three or more items were considered the minimum for retention for factors.

The factor structure was examined in analyses with the entire sample (n = 1047), and in analyses restricted to the combined GP and adult nurse professional groupings (n = 548) in order to explore the dimensions underlying the R-DAQ within a generalist primary care professional group.

#### Convergent validity

Convergent validity was assessed performing item-scale correlations corrected for overlaps. Correlations were calculated using Pearson’s product moment correlation coefficient, and acceptable corrected item-total correlations were those ≥0.2 [40].

#### Internal consistency

Internal consistency was assessed by Cronbach’s alpha values, conducted for the total scale and for the subscales that emerged from the factor analysis. This test of the interrelatedness of items ranges from 0 to 1, with an acceptable range usually considered to be between 0.70 to 0.90 [40], though a lower margin of 0.6 is sometimes applied [41].

#### Test-retest reliability

An independent sample of 38 health professionals completed the measure twice with a retest interval of 7 to 19 days (*M* = 11.5 days). Test-retest reliability was measured by the intraclass correlation coefficient using a two factor mixed effects model and type consistency, calculated as the ratio of the sums of various variance component estimates and defined with values between 0 and 1 with cut-offs ≤0.40 for *poor*, 0.41–0.59 *fair*, 0.60–0.74 *good*, ≥0.75 *excellent* [42].

#### Floor and ceiling effects

If a significant proportion of people have scores at the bottom (floor) or top (ceiling) of the range of possible scores, then the potential responsiveness of tool will be impaired as it will not necessarily measure change. Floor and ceiling effects were assessed by examining response patterns for each of the factors derived from exploratory factor analysis. Scores were graphed as a histogram and the distribution of scores inspected; the percentage of individuals with the lowest and highest possible score in each of the factors was recorded, and values greater than 20% were considered as floor and ceiling effects [43].

#### Readability

This was assessed using the Flesch Reading Ease score and Flesch-Kincaid Grade level functions (available within Microsoft Word), widely-used tests which evaluate readability based on syllables per word and words per sentence [44]. Scores for the former are typically noted from 0–100 (though theoretically there is no lower bound and the upper limit may be around 120), with higher scores indicating greater ease of understanding, and scores of 60–70 representing wording easily understood by 13- to 15-year-old students, whereas scores between 0 and 30 indicate that the text is better suited to graduate level readers. The Flesch-Kincaid Grade level scale provides the readability level in relation to the US educational grade to which the material is most appropriate.

## Results

### Development phase

A total of 24 of the 32 experts (75%) who were contacted consented to involvement in the Delphi study and participated in the first round, 22 completed the second round, and 21 the final third round. The experts panellists involved in this exercise comprised academic and clinical nurses (n = 8), GPs (n = 7), psychiatrists (n = 4), psychologists (n = 2), social scientists (n = 2) and pharmacists (n = 1), and were based in: the UK (n = 15), the USA (3), Australia (2), Belgium (1), Finland (1), Estonia (1), and Italy (1).

Of the 26 candidate items proposed in the first round, 23 items achieved greater than 70% endorsement at the first round. In the second round these 23 items and 14 proposed further items were included together several suggestions for re-phrasing; and by the final third round 30 of a set of 40 items achieved the proposed item-level CVI threshold mean value of 0.78 (the scores of these retained items ranged between 1.0 and 0.79, whereas the CVI scores for the rejected items ranged between 0.2 and 0.75). The scale-level CVI determined by summing the CVI scores for items meeting item-level threshold, and dividing by the number of items, was 0.96, meeting the level of 0.90 set as the standard for this index.

### Testing phase

#### Sample

1193 responses to the invitation to participate in the testing of the R-DAQ were received during the designated testing period (October 2011 to February 2012), an overall response rate of 16%. 146 of these were removed from the analysis because the questionnaire was not completed (n = 124) or the individual respondent was not a health professional (n = 22). All respondents were from the UK, with the exception of one from Finland and four from Italy. 90% of respondents were nurses - grouped in line with current UK registration as either Adult, incorporating mostly nurses working in GP practices as part of the primary care team (practice nurses), or Mental (mental health nurses). Other health professionals who responded were GPs, counsellors, psychological therapists, and a consultant psychiatrist (Table 1).

**Table 1:** **Respondents by professional group**

**Profession**	**n**	**%**
GP	37	3.5
Nurse (Adult)	511	48.8
Nurse (Mental)	427	40.8
Other Health Professional	72	6.9
Total	1047	100.0

### Descriptives

A full range of responses (categories one through to five) was evident for all 30 items in the testing version of the R-DAQ. Given that the R-DAQ contains discrete variables, the item distributions were expected to demonstrate some degree of non-normality. Consistent with this expectation, most (28) of the initial 30 items exhibited mild to moderate negative skewness (between −0.12 and −1.4, for all but two items: items 22 and 16–3.4 and −1.6 respectively). Mild to moderate negative kurtosis (−0.004 to −1.2) was evident for 12 items; and 3 items which were retained [items 5; 9; 10] exhibited distributions with excess positive kurtosis between 2.0 and 3.0, whilst for two items the values were 5.0 [item 16] and 16.4 [item 22]. Examination of Q-Q plots and detrended normal Q-Q plots indicated that deviation from normality was not marked.

Mean values for the individual items ranged from 4.77 (95% C.I. 4.73 to 4.80) (SD = 0.55) [item 22] to 3.29 (95% C.I. 3.21 to 3.37) (SD = 1.32) [item 8]. Following scale reduction to a 22-item scale, participants’ total scores ranged between 57 and 110; the mean value was 87.74 (95% C.I. 87.14 to 88.34) (median = 88.0; SD = 9.84).

#### Exploratory factor analysis

Principal axis factoring was undertaken to explore latent constructs and to assess the dimensionality of this scale. Prior to conducting this analysis, tests of the suitability of the data were conducted, the overall KMO statistic was 0.87, and Bartlett’s Test of Sphericity was significant (p < 0.001). The correlation matrix showed no extreme multicollinearity or singularity: the majority of items exhibited correlations >0.3 the highest correlation between items was 0.78, and the determinant of the correlation matrix was greater than 0.001. The individual measures of sampling adequacy for each of the 30 items were examined in the anti-image of the correlation matrix, with most items above 0.9 (marvellous) or 0.8 (meritorious); five items were between 0.7 and 0.8 (middling), and four items were between 0.6 and 0.7 (mediocre). Items with the lowest individual KMO statistic were considered for dropping from the analysis, with judgments additionally made on the basis of the magnitudes of communalities and factor loadings derived from factor analyses. Eight items were removed from the initial 30-item scale, on the basis of low communalities (<0.2), low individual measures of sampling adequacy (<0.7), and low factor loadings (<0.3), together with judgements about the interpretability and theoretical clarity within emerging factor structures.

**Figure 1:**
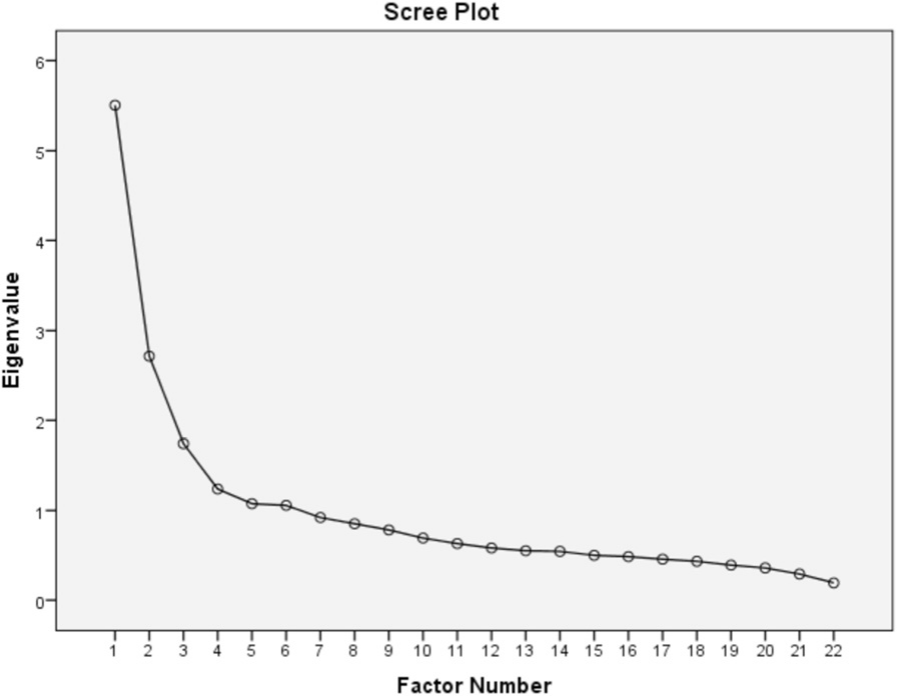
**Scree plot for 22-item R-DAQ.**

The criteria for determining the number of factors were multi-faceted, and took into account the Kaiser criterion, scree plots and amount of variance explained by potential models, but with an emphasis on interpretability. Initially, for the 30-item scale, 8 factors displayed eigen values above 1.0, explaining 56.8% variance. The scree plot indicated a 3- or 4-factor solution (Figure 1), and these factor solutions was explored, with item performance iteratively evaluated with removal of items conducted in a step-by-step process. A three-factor solution generated the most comprehensible and parsimonious factor structure and was considered optimal. The final factor analytic solution comprised 22 items. It provided a KMO measure of sampling adequacy of 0.87 and explained 45.3% of the variance (Tables 2 and 3).

**Table 2:** **Factor analysis pattern matrix, R-DAQ, total sample**

**R-DAQ items**	**Factor**		
	**1**	**2**	**3**
7: I feel confident in assessing depression in patients	**.896**	-.043	.016
17: I feel confident in assessing suicide risk in patients presenting with depression	**.856**	-.042	.019
1: I feel comfortable in dealing with depressed patients’ needs	**.789**	.034	.044
15: My profession is well trained to assist patients with depression	**.777**	-.087	-.076
8: I am more comfortable working with physical illness than with mental illnesses like depression (reversed)	**.715**	.069	-.083
11: My profession is well placed to assist patients with depression	**.617**	.011	.063
19: It is rewarding to spend time looking after depressed patients	**.583**	.078	.128
20: Becoming depressed is a natural part of adolescence (reversed)	-.106	**.646**	-.015
12: Becoming depressed is a way that people with poor stamina deal with life difficulties (reversed)	-.019	**.639**	.082
18: Depression reflects a response which is not amenable to change (reversed)	.080	**.570**	.008
5: One of the main causes of depression is a lack of self-discipline and will-power (reversed)	.008	**.564**	.093
6: Depression treatments medicalise unhappiness (reversed)	-.073	**.532**	.014
9: Becoming depressed is a natural part of being old (reversed)	-.119	**.525**	.009
21: There is little to be offered to depressed patients who do not respond to initial treatments (reversed)	.136	**.478**	-.041
3: Psychological therapy tends to be unsuccessful with people who are depressed (reversed)	.268	**.360**	-.020
13: Once a person has made up their mind about taking their own life no one can stop them (reversed)	.046	**.348**	-.037
4: Antidepressant therapy tends to be unsuccessful with people who are depressed (reversed)	.151	**.302**	.018
22: Anyone can suffer from depression	.006	.068	**.533**
16: Recognising and managing depression is often an important part of managing other health problems	.056	.051	**.521**
14: People with depression have care needs similar to other medical conditions like diabetes, COPD or arthritis.	.004	-.081	**.460**
2: Depression is a disease like any other (e.g. asthma, diabetes)	-.077	-.028	**.454**
10: All health professionals should have skills in recognising and managing depression	.088	.081	**.388**

Extraction Method: Principal Axis Factoring. Rotation Method: Oblimin with Kaiser Normalization.

Rotation converged in 5 iterations. Emboldened figures show factor loading in relation to each item and factor.

**Table 3:** **Factor analysis pattern matrix, R-DAQ, restricted sample**

**R-DAQ items**	**Factor**		
	**1**	**2**	**3**
20: Becoming depressed is a natural part of adolescence (reversed)	**.635**	.062	-.046
9: Becoming depressed is a natural part of being old (reversed)	**.579**	.109	-.039
12: Becoming depressed is a way that people with poor stamina deal with life difficulties (reversed)	**.557**	.075	.188
6: Depression treatments medicalise unhappiness (reversed)	**.527**	.112	.033
21: There is little to be offered to depressed patients who do not respond to initial treatments (reversed)	**.526**	-.191	-.078
5: One of the main causes of depression is a lack of self-discipline and will-power (reversed)	**.461**	.116	.225
18: Depression reflects a response which is not amenable to change (reversed)	**.434**	-.120	.108
13: Once a person has made up their mind about taking their own life no one can stop them (reversed)	**.367**	-.073	-.073
3: Psychological therapy tends to be unsuccessful with people who are depressed (reversed)	**.340**	-.233	.083
4: Antidepressant therapy tends to be unsuccessful with people who are depressed (reversed)	**.328**	-.145	.011
7: I feel confident in assessing depression in patients	-.030	**-.799**	.046
17: I feel confident in assessing suicide risk in patients presenting with depression	-.070	**-.733**	.016
1: I feel comfortable in dealing with depressed patients’ needs	.114	**-.673**	.019
8: I am more comfortable working with physical illness than with mental illnesses like depression (reversed)	.079	**-.579**	.011
15: My profession is well trained to assist patients with depression	-.129	**-.529**	-.104
19: It is rewarding to spend time looking after depressed patients	.099	**-.519**	.137
11: My profession is well placed to assist patients with depression	.039	**-.412**	.052
22: Anyone can suffer from depression	.039	.085	**.606**
16: Recognising and managing depression is often an important part of managing other health problems	-.009	-.106	**.555**
2: Depression is a disease like any other (e.g. asthma, diabetes)	-.003	.035	**.468**
10: All health professionals should have skills in recognising and managing depression	.081	-.101	**.437**
14: People with depression have care needs similar to other medical conditions like diabetes, COPD or arthritis.	-.064	-.025	**.430**

Extraction Method: Principal Axis Factoring. Rotation Method: Oblimin with Kaiser Normalization.

Rotation converged in 7 iterations. Emboldened figures show factor loading in relation to each item and factor.

### Internal consistency and convergent validity

Cronbach’s α for the internal consistency of the 22 item scale was 0.84 with corrected item-total correlations between 0.24 and 0.66 for 20 items; two items with inadequate item-total correlations (0.10 and 0.13) were also retained because of their factor loadings and fit with other factor items. When analysis was restricted to GPs and adult nurses, the corrected item-total correlations for all items exceeded 0.20 (range 0.21 to 0.53), and the internal consistency of the 22-item scale was 0.80 within this participant sub-group (Table 4).

**Table 4:** **Internal consistency and item-total correlation for 22-tem R-DAQ for total sample, and restricted to adult nurses and GPs**

**Revised Depression Attitude Questionnaire (R-DAQ) Items (22)**			**Corrected item-total correlation**		**Cronbach’s α if item deleted**	
**Factor**	**Item**		**Total sample**	**Restricted sample**	**Total sample**	**Restricted sample**
1	7	I feel confident in assessing depression in patients	.658	.514	.818	.783
	1	I feel comfortable in dealing with depressed patients’ needs	.656	.530	.820	.782
	17	I feel confident in assessing suicide risk in patients presenting with depression	.631	.434	.819	.788
	19	It is rewarding to spend time looking after depressed patients	.571	.499	.825	.786
	8	I am more comfortable working with physical illness than with mental illnesses like depression (reversed)	.554	.422	.824	.789
	11	My profession is well placed to assist patients with depression	.527	.338	.825	.794
	15	My profession is well trained to assist patients with depression	.506	.211	.826	.802
2	3	Psychological therapy tends to be unsuccessful with people who are depressed (reversed)	.448	.436	.830	.789
	18	Depression reflects a response which is not amenable to change (reversed)	.437	.407	.830	.790
	12	Becoming depressed is a way that people with poor stamina deal with life difficulties (reversed)	.425	.394	.831	.791
	21	There is little to be offered to depressed patients who do not respond to initial treatments (reversed)	.420	.449	.830	.788
	5	One of the main causes of depression is a lack of self-discipline and will-power (reversed)	.408	.314	.831	.795
	4	Antidepressant therapy tends to be unsuccessful with people who are depressed (reversed)	.341	.327	.834	.794
	20	Becoming depressed is a natural part of adolescence (reversed)	.338	.345	.834	.794
	6	Depression treatments medicalise unhappiness (reversed)	.303	.273	.835	.797
	13	Once a person has made up their mind about taking their own life no one can stop them (reversed)	.259	.261	.837	.798
	9	Becoming depressed is a natural part of being old (reversed)	.246	.274	.837	.797
3	10	All health professionals should have skills in recognising and managing depression	.271	.344	.836	.794
	16	Recognising and managing depression is often an important part of managing other health problems	.251	.324	.836	.795
	22	Anyone can suffer from depression	.237	.241	.837	.798
	14	People with depression have care needs similar to other medical conditions like diabetes, COPD or arthritis	.134	.205	.843	.801
	2	Depression is a disease like any other (e.g. asthma, diabetes)	.102	.227	.846	.800

The first factor, *professional confidence in depression care,* was comprised of 7 items (3 derived from the original DAQ) concerning feeling comfortable, confident, and well-trained as an individual practitioner and member of a profession providing depression care. The Cronbach’s α value was 0.90, with values for the corrected item-subscale correlation of its items ranging from 0.59 to 0.83. When restricted to GPs and adult nurses, Cronbach’s α was 0.81, and corrected item-subscale correlations were between 0.38 and 0.70. This factor explained 25.0% of the total variance within the factor structure model.

The 10 items in the second factor concerning *therapeutic optimism about depression* were comprised of reverse scored pessimistic and deterministic statements about depression and its treatment, 6 of which were derived from the original DAQ. These items had a Cronbach’s α coefficient of 0.78, with corrected item-subscale correlations between 0.33 and 0.55. When restricted to GPs and adult nurses, Cronbach’s α was 0.76, and corrected item- subscale correlations were 0.30-0.51. This factor explained 12.3% of the total scale variance.

Cronbach’s α for the third factor (5 items), about a *generalist perspective about depression occurrence, recognition and management* was 0.57 with corrected item- subscale correlations ranging from 0.29 to 0.38 (when restricted to GPs and adult nurses, Cronbach’s α was 0.62, and corrected item- subscale correlations were 0.34 to 0.43). This factor explained 7.9% of the total variance. None of these items originated from the DAQ.

#### Test-retest reliability

The 22-item R-DAQ was completed by 38 health professionals (16 adult nurses and 15 student nurses, 5 GPs, a final-year medical student, and a clinical psychologist) on two occasions with a retest interval of 7 to 19 days (*M* = 11.5 days). The test–retest reliability measured by the intraclass correlation coefficient (calculated as the ratio of the sums of various variance component estimates and defined with values between 0 and 1) was 0.62 (95% C.I. 0.37 to 0.78), indicating good or substantial agreement.

#### Floor and ceiling effects

The frequency of scores for the derived factors was examined by producing histograms and inspecting the distribution of scores. The data for the three factors were negatively skewed with higher scores most common (i.e. a higher frequency of endorsing statements describing positive attitudes and confidence). Response patterns were examined for the attitude factors and showed this distribution was most apparent for the third factor *generalist perspective about depression occurrence, recognition and management*, for which 12.3% of participants scored 5.00 (mean score 4.29, SD = 0.50). For the second factor, *therapeutic optimism*, 2% scored 5.00 (mean score 4.09, SD = 0.48), whilst for the first factor *professional confidence in managing depression* the mean score was 3.63 (SD = 0.90), with 4% scoring 5.00. These results indicate that the R-DAQ is likely to be responsive to change with responses unimpaired by floor or ceiling effects.

#### Readability

The readability evaluation of the 22-item R-DAQ provided a Flesch Reading Ease score of 46.7, and a Flesch-Kincaid Grade level of 9.4, indicating that these items are likely to be understandable to a typical 14–15 year-old student. The original 20-item DAQ that this version seeks to replace has a Flesch Reading Ease score of 19.6, and a Flesch-Kincaid Grade level of 13.9, which indicates a far less readable text, written in a way best suited to graduates.

## Discussion

This study sought to build upon a prior review and pooled analysis of the existing DAQ measure of health professionals’ attitudes to depression. Deficits in this original instrument coupled with the importance of better understanding and measuring the attitude responses of clinical staff to depression, provided the motivation for this work. The R-DAQ scale is provided in Additional file 1.

### Strengths and limitations

The major strengths of the current study are the process of measure development based on detailed review and examination of the psychometric properties of the original DAQ together with review and consideration of related instruments; the involvement of a representative expert panel group to assist item selection and generation and to assess content validity; and the relatively broad assessment of the psychometric properties of the revised (R-DAQ) measure with a sufficiently sized sample of health care professionals.

The key benefits of this new measure are its improved psychometric properties and readability, which make it more appropriate for use in a range of primary care and other health settings where health professionals are likely to encounter depression and assist in its care, whereas the original DAQ has several important weaknesses related to its over-extensive content coverage, complex wording, and development within a specific setting. Nonetheless, the R-DAQ incorporates a number of items (9 items) from the original 20-item scale which will allow comparison of findings at item level with existing DAQ data. The phrasing of the scale items is balanced between positive and negative perspectives: 11 of the 22 items in the final scale are negatively framed, requiring reverse scoring. The potential responsiveness of the R-DAQ was gauged using floor and ceiling effects for the three factor sub-scales, and indicated its ability to detect change in the population for which it was designed.

There are a number of study limitations. Firstly, the study sample was chosen by convenience: rather than a random group of the target audience of health professionals who are involved in depression management, it was comprised of a self-selected group of mostly UK nurses who were recruited through a specific organisation. As such the study participants may not be representative of the variety of settings and of professional groupings for which this scale is designed. In particular, the proportion of GP respondents is low which may limit the applicability of the R-DAQ to this key professional group. The analysis restricted to a generalist primary care professional group (combined GPs and adult nurses) may in part address this limitation.

Secondly, the response rate to the survey invitation was low (though comparable with similar studies), which is an additional potential source of selection bias which limits the external validity (generalizability) of findings. A systematic review of survey response rates for differing administration modes indicates mean rates of 45% for mail surveys and 34% for Web surveys, with reduced response significantly associated with professional respondents compared with other population types, and possibly also be related to the absence of reminders or incentives [45].

Thirdly, because of the electronic survey software design which required responses to all items, there is no information about the extent or patterns of missing data that would apply with the R-DAQ. Such data are useful in understanding and evaluating measure acceptability by evaluating the frequency of missing responses to individual items.

### Applications

The R-DAQ is designed to identify and quantify the attitudes of health professionals to depression: this is important in determining the relative need for and impact of a wide range of interventions that may influence the recognition, support and treatment for this common and disabling condition. Understanding the effects of approaches such as education and training, guideline implementation drives, incentives, mass media campaigns, and organisational changes affecting the context and environment in which depressed patients consult and the availability of the necessary resources for management, is crucial to improving service quality; and measuring changes in care providers’ attitudes is part of overall evaluation which can assist in clarifying mechanisms of effect.

The constitution and focus of the expert panel and of the selection of the participants involved in the psychometric testing of the R-DAQ indicate its appropriateness and utility for use with health professionals across a range of disciplines and care settings. This is important because there is widespread recognition that depression identification and management are a key part of the role of health professionals in primary, community and general medical care as well as of mental health specialists [46].

### Implications for future research

This study provides a robust initial basis for further validation of the R-DAQ with samples representative of the wider health care workforce. As well as extending the testing of this tool (ideally with greater numbers of GPs and respondents from settings additional to the UK), future research should explore variables that may influence responses such as responder profession, gender, type and duration of experience, and setting of practice.

The R-DAQ describes and quantifies health professionals’ attitudes to depression with psychometric properties sufficient for its use to examine this important provider characteristic. Research indicates that the attitudes of health professionals to depression and other mental health problems is variable and may incorporate negative and unhelpful views. Determining the best, most effective ways of informing clinicians’ attitudes is an educational priority, and the R-DAQ will be a useful tool in evaluating programmes developed for this purpose.

## Conclusions

Attitudes play a key part in the behaviour both of the public and of heath care providers in relation to depression and other mental illnesses. Research indicates that although improvements are evident over the past 20 years within the general population in the understanding, tolerance and greater acceptance of professional help for mental health problems, some views, such as that mental illness is related to a lack of will-power or self-discipline, or that people with mental illness are prone to violence, seem unchanged or increasingly evident [8,47]. Some of these negative and stigmatising perspectives that persist within the wider population appear evident among health professionals; and examining and measuring attitudes is a necessary part of work to extend the therapeutic confidence and positive, supportive potential of service providers.

Our study produced a revised version of the DAQ measure that has been used by researchers since its development in 1992. The R-DAQ consists of 22 items and incorporates various dimensions encompassing professional confidence, therapeutic optimism, and views about generalist or specialist perspectives pertinent to depression and its care. The high prevalence of depression, the extent of associated disability and the likelihood of its co-occurrence with other medical conditions provide a strong rationale for developing the capability and extending the competence of health care providers, particularly within the primary care workforce. This revised measure demonstrates face, content and construct validity established by appropriate methods, and adequate internal consistency and test-retest reliability; the modest number of items and readability level indicate that it can be used with health care professionals in the busy environments in which they provide care.

## Additional file

Additional file 1:
**Revised Depression Attitude Questionnaire (R-DAQ).**

## References

[1] WHO (2012). Depression: a global public health concern.

[2] Waraich P, Goldner EM, Somers JM, Hsu L (2004). Prevalence and incidence studies of mood disorders: a systematic review of the literature. Can J Psychiatry.

[3] Hawton K, van Heeringen K (2009). Suicide. Lancet.

[4] World Health Organization (2008). The global burden of disease: 2004 update.

[5] Moussavi S, Chatterji S, Verdes E, Tandon A, Patel V, Ustun B (2007). Depression, chronic diseases, and decrements in health: results from the World Health Surveys. Lancet.

[6] Menchetti M, Murri MB, Bertakis K, Bortolotti B, Berardi D (2009). Recognition and treatment of depression in primary care: effect of patients’ presentation and frequency of consultation. J Psychosom Res.

[7] Mitchell AJ, Vaze A, Rao S (2009). Clinical diagnosis of depression in primary care: a meta-analysis. Lancet.

[8] Schomerus G, Schwahn C, Holzinger A, Corrigan PW, Grabe HJ, Carta MG (2012). Evolution of public attitudes about mental illness: a systematic review and meta-analysis. Acta Psychiatr Scand.

[9] Dumesnil H, Cortaredona S, Verdoux H, Sebbah R, Paraponaris A, Verger P (2012). General practitioners’ choices and their determinants when starting treatment for major depression: a cross sectional, randomized case-vignette survey. PLoS One.

[10] Ross S, Moffat K, McConnachie A, Gordon J, Wilson P (1999). Sex and attitude: a randomized vignette study of the management of depression by general practitioners. Br J Gen Pract.

[11] Kendrick T, King F, Albertella L, Smith PW (2005). GP treatment decisions for patients with depression: an observational study. Br J Gen Pract.

[12] Dowrick C, Gask L, Perry R, Dixon C, Usherwood T (2000). Do general practitioners’ attitudes towards depression predict their clinical behaviour?. Psychol Med.

[13] Botega N, Mann A, Blizard R, Wilkinson G (1992). General practitioners and depression - first use of the depression attitude questionnaire. Int J Meth Psych Res.

[14] Kerr M, Blizard R, Mann A (1995). General practitioners and psychiatrists: comparison of attitudes to depression using the depression attitude questionnaire. Br J Gen Pract.

[15] Haddad M, Walters P, Tylee A (2007). District nursing staff and depression: a psychometric evaluation of Depression Attitude Questionnaire findings. Int J Nurs Stud.

[16] Haddad M, Butler GS, Tylee A (2010). School nurses’ involvement, attitudes and training needs for mental health work: A UK-wide cross-sectional study. J Adv Nurs.

[17] Scheerder G, De Coster I, Van Audenhove C (2009). Community pharmacists’ attitude toward depression: a pilot study. Res Social Adm Pharm.

[18] Waller R, Hillam JC (2000). Assessment of depression in older medical inpatients: practice, attitudes and the effect of teaching. Aging Ment Health.

[19] Haddad M, Menchetti M, Walters P, Norton J, Tylee A, Mann A (2012). Clinicians’ attitudes to depression in Europe: a pooled analysis of depression attitude questionnaire findings. Fam Pract.

[20] Botega NJ, Silveira GM (1996). General practitioners attitudes towards depression: a study in primary care setting in Brazil. Int J Soc Psychiatry.

[21] Richards JC, Ryan P, McCabe MP, Groom G, Hickie IB (2004). Barriers to the effective management of depression in general practice. Aust N Z J Psychiatry.

[22] James BO, Jenkins R, Lawani AO, Omoaregba JO (2012). Depression in primary care: the knowledge, attitudes and practice of general practitioners in Benin City, Nigeria. S Afr Fam Pract.

[23] Ohtsuki T, Kodaka M, Sakai R, Ishikura F, Watanabe Y, Mann A (2012). Attitudes toward depression among Japanese non-psychiatric medical doctors: a cross-sectional study. BMC Res Notes.

[24] Hsu CC, Sandford BA (2007). The Delphi technique: making sense of consensus. Pract Assess Res Eval.

[25] Polit DF, Beck CT (2006). The content validity index: are you sure you know what’s being reported? Critique and recommendations. Res Nurs Health.

[26] Priest RG, Vize C, Roberts A, Roberts M, Tylee A, MORI Poll: Defeat Depression Campaign. 1992, in (1996). Lay people’s attitudes to treatment of depression: results of opinion poll for Defeat Depression Campaign just before its launch. BMJ.

[27] Scheerder G, Audenhove CV, Arensman E, Bernik B, Giupponi G, Horel AC (2011). Community and health professionals’ attitude toward depression: a pilot study in nine EADD countries. Int J Soc Psychiatry.

[28] Mehta N, Kassam A, Leese M, Butler G, Thornicroft G (2009). Public attitudes towards people with mental illness in England and Scotland, 1994–2003. Br J Psychiatry.

[29] Taylor SM, Dear MJ (1981). Scaling community attitudes toward the mentally ill. Schizophr Bull.

[30] Lynn MR (1986). Determination and quantification of content validity. Nurs Res.

[31] Costello AB, Osborne JW (2005). Best practices in exploratory factor analysis: four recommendations for getting the most from your analysis. Pract Assess Res Eval.

[32] Gaskin CJ, Happell B (2014). On exploratory factor analysis: a review of recent evidence, an assessment of current practice, and recommendations for future use. Int J Nurs Stud.

[33] Lusk C, Delclos GL, Burau K, Drawhorn DD, Aday LA (2007). Mail versus internet surveys: determinants of method of response preferences among health professionals. Eval Health Prof.

[34] Inc SPSS (2006). SPSS advanced models 15.0.

[35] Kaiser HF (1974). Computing measures of simplicity of fit for loadings in factor-analytically derived scales. Psychometrika.

[36] Childs D (1970). The essentials of factor analysis.

[37] Bartlett MS (1954). A note on multiplying factors for various chi square approximations. J R Stat Soc.

[38] Cattell RB (1966). The scree test for the number of factors. Multivar Behav Res.

[39] Tabachnick BG (2013). Using multivariate statistics.

[40] Streiner DL (2008). Health measurement scales: a practical guide to their development and use.

[41] Stanley RM, Ridley K, Olds TS, Dollman J (2014). Development and psychometric properties of the Y-PASS questionnaire to assess correlates of lunchtime and after-school physical activity in children. BMC Public Health.

[42] Mcgraw KO, Wong SP (1996). Forming inferences about some intraclass correlation coefficients. Psychol Methods.

[43] McHorney CA, Ware JE, Lu JF, Sherbourne CD (1994). The MOS 36-item Short-Form Health Survey (SF-36): III. Tests of data quality, scaling assumptions, and reliability across diverse patient groups. Med Care.

[44] Flesch R (1948). A new readability yardstick. J Appl Psychol.

[45] Shih T, Fan X (2008). Comparing response rates from web and mail surveys: a meta-analysis. Field Methods.

[46] Haddad M, Walters P, Tylee A (2009). Mood disorders in primary care. Psychiatr.

[47] TNS-BRMB: Attitudes to mental illness 2012 Research report, prepared for the Time to Change 2013. www.mind.org.uk/..118308-attitudes-to-mental-illness-2012-report-v6.docx. 2013.

