# Supplementary material for: The development and psychometric properties of a measure of clinicians’ attitudes to depression: the revised Depression Attitude Questionnaire (R-DAQ)
Source: BMC Psychiatry. 2015 Feb 5;15:7. doi: 10.1186/s12888-014-0381-x (PMC4321322; doi:10.1186/s12888-014-0381-x)
Supplement: Additional file 1: — Revised Depression Attitude Questionnaire (R-DAQ). [file 12888_2014_381_MOESM1_ESM.docx]

Revised Depression Attitude Questionnaire (R-DAQ), Haddad et al 2014

|  | Please read the statement and tick/click the box that relates best to your personal opinion | Strongly disagree | Disagree | Neither disagree nor agree | Agree | Strongly agree |
| --- | --- | --- | --- | --- | --- | --- |
| 1 | **I feel comfortable in dealing with depressed patients’ needs** |  |  |  |  |  |
| 2 | **Depression is a disease like any other (e.g. asthma, diabetes)** |  |  |  |  |  |
| 3 | **Psychological therapy tends to be unsuccessful with people who are depressed** |  |  |  |  |  |
| 4 | **Antidepressant therapy tends to be unsuccessful with people who are depressed** |  |  |  |  |  |
| 5 | **One of the main causes of depression is a lack of self-discipline and will-power** |  |  |  |  |  |
| 6 | **Depression treatments medicalise unhappiness** |  |  |  |  |  |
| 7 | **I feel confident in assessing depression in patients** |  |  |  |  |  |
| 8 | **I am more comfortable working with physical illness than with mental illnesses like depression** |  |  |  |  |  |
| 9 | **Becoming depressed is a natural part of being old** |  |  |  |  |  |
| 10 | **All health professionals should have skills in recognising and managing depression** |  |  |  |  |  |
| 11 | **My profession is well placed to assist patients with depression** |  |  |  |  |  |
| 12 | **Becoming depressed is a way that people with poor stamina deal with life difficulties** |  |  |  |  |  |
| 13 | **Once a person has made up their mind about taking their own life no one can stop them** |  |  |  |  |  |
| 14 | **People with depression have care needs similar to other medical conditions like diabetes, COPD or arthritis** |  |  |  |  |  |
| 15 | **My profession is well trained to assist patients with depression** |  |  |  |  |  |
| 16 | **Recognising and managing depression is often an important part of managing other health problems** |  |  |  |  |  |
| 17 | **I feel confident in assessing suicide risk in patients presenting with depression** |  |  |  |  |  |
| 18 | **Depression reflects a response which is not amenable to change** |  |  |  |  |  |
| 19 | **It is rewarding to spend time looking after depressed patients** |  |  |  |  |  |
| 20 | **Becoming depressed is a natural part of adolescence** |  |  |  |  |  |
| 21 | **There is little to be offered to depressed patients who do not respond to initial treatments** |  |  |  |  |  |
| 22 | **Anyone can suffer from depression** |  |  |  |  |  |
